# ‘I'll put up with things for a long time before I need to call anybody’: Face work, the Total Institution and the perpetuation of care inequalities

**DOI:** 10.1111/1467-9566.13435

**Published:** 2022-01-25

**Authors:** Jo Hope, Lisette Schoonhoven, Peter Griffiths, Lisa Gould, Jackie Bridges

**Affiliations:** ^1^ National Institute for Health Research (NIHR) Applied Research Collaboration (ARC) Wessex Southampton UK; ^2^ 7423 Faculty of Environmental and Life Sciences School of Health Sciences University of Southampton Southampton UK; ^3^ 8125 Julius Center for Health Sciences and Primary Care University Medical Center Utrecht Utrecht University Utrecht The Netherlands; ^4^ Department of Surgery and Cancer Faculty of Medicine Institute of Global Health Innovation Imperial College London London UK

**Keywords:** fundamental care, Goffman, hospitals, missed care, nursing, patient experience, personalisation

## Abstract

Failures in fundamental care (e.g. nutrition or pain‐relief) for hospitalised patients can have serious consequences, including avoidable deaths. Policy rhetoric of ‘shared decision‐making’ fails to consider how structural constraints and power dynamics limit patient agency in nursing staff‐patient interactions. Goffman's concepts of face work, the presentation of self and the Total Institution shaped our analysis of interview and focus group data from hospital patients. Patients avoided threatening ‘good’ patient and staff face by only requesting missed care when staff face was convincing as ‘caring’ and ‘available’ (‘engaged’). Patients did not request care from ‘distracted’ staff (‘caring’ but not ‘available’), whilst patient requests were ignored in Total Institution‐like ‘dismissive’ interactions. This meant patients experienced missed care with both ‘distracted’ and ‘dismissive’ staff. Patients with higher support needs were less able to carry out their own missed care to protect staff face, so experienced more serious care omissions. These findings show that many elements of the Total Institution survive in modern healthcare settings despite attempts to support individualised care. Unless nursing staff can maintain face as ‘engaged’ (despite organisational constraints that can reduce their capacity to do so) patient participation in care decisions will remain at the level of rhetoric.

## INTRODUCTION

Missed fundamental care in hospital (personal cleansing, dressing, toileting needs, eating and drinking, rest and sleep, mobility, comfort and safety: Feo et al., 2018) can have serious consequences including avoidable deaths (Francis, 2013; Heslop et al., 2013; Parliamentary and Health Service Ombudsman and Local Government Ombudsman, 2009). Kalisch et al.(2014) argued that patients have a key role in flagging missed care. However, an emerging literature shows patients can be reluctant to ask for care for fear of being labelled difficult and receiving poorer care. Current conceptualisations of nurse–patient interactions neglect the patient's role and fail to encompass the structural constraints on patient–nurse interactions, including the need to present as a ‘good patient’. Our study explores how patients manage fundamental care requests in hospital, using the lens of Goffman's concepts of face work, impression management and the Total Institution (Goffman, 1959, 1961, 1967).

Missed care in hospitals is common and when patient need exceeds labour resources, medical care tends to be prioritised, with implicit rationing of both emotional support and aspects of fundamental care (Jones et al., 2015; Kalisch, 2006; Kalisch et al., 2009). Bail and Grealish (2016) conceptualise this as a ‘failure to maintain’, which disproportionately impacts older patients including those with dementia, leading to functional, cognitive and physical issues at discharge. This might also explain why older people with cognitive impairments appear to be more vulnerable to the adverse consequences of low nurse staffing, including death, whilst in hospital (Fogg et al., 2021).

Nurse–patient interactions can be constrained by a range of organisational factors including structure, culture, bureaucratic constraints, staffing, pressure, role demands (Fleischer et al., 2009) and ward physical layout (Donetto et al., 2017). These constraints can reduce capacity to provide care to the quality that staff would prefer (Bridges et al., 2017). This lack of capacity to provide individualised, empathic care could be one mechanism through which lowered staffing levels are related to an increase in negative care interactions (Bridges et al., 2019) as well as explaining how fundamental care is missed.

Patient involvement in health care is well established as leading to better health outcomes and reducing costs (Hibbard & Greene, 2013) and patients could have a key role in flagging missed care (Kalisch et al., 2014). However, our understanding of the patient role in the nurse–patient interaction remains under‐conceptualised (Fleischer et al., 2009). We know from the research literature that patients have strong ideas about what quality care involves (e.g. Irurita, 1999), but that their ability to make informed choices and be involved in decision‐making can be limited by nurses withholding information or limiting involvement in decision‐making (Crispin et al., 2017; Gremigni et al., 2016; Henderson, 2008; Tobiano et al., 2015). Conceptually, we need to develop a better understanding of how a lack of availability, empathy and approachability of staff reduces involvement in care decisions, with patients feeling unable to request physical support required to carry out fundamental care (Kitson et al., 2013), and in the worst cases experiencing care interactions that leave them feeling dehumanised, objectified, disempowered or devalued (Bridges et al., 2020; Coyle and Williams, 2001). Patients with more complex needs are perceived as intrinsically less rewarding to care for (Arnetz et al., 2016; Bridges et al., 2020; Maben et al., 2012) or disruptive or difficult when they resist or slow down care tasks (Featherstone & Northcott, 2020). Patient capability and condition have also been related to lowered participation in decision‐making around physical care in medical wards in Denmark and Iran (Lomborg & Kirkevold, 2008; Soleimani et al., 2010). Patients (who are able to) will work to avoid being labelled difficult, for example by not ‘interrupting’ nursing staff who appeared rushed, which involves significant emotional labour (Maben et al., 2012).

These empirical findings are in stark contrast to conceptualisations of patient‐staff interactions in current UK policy of ‘shared decision‐making’. This rests on the assumption of empowered healthcare professionals who ‘fully explore’ care options and patients who ‘feel supported and empowered to make informed choices and reach a shared decision about care’ (NHS England, 2019). Such conceptions of person‐centred care lean towards the logic of choice described by Mol (2008), where patients are framed as consumers responsible for making a choice from an array of options, as opposed to a logic of care that is collaborative, experimental and involves ‘inventive doctoring’ to meet the patient's needs. Similarly, nursing literature exploring nurse–patient interactions has rarely engaged with sociological work (Fleischer et al., 2009; Stoddart, 2012). This means little consideration has been given to the structural constraints, power dynamics and health inequalities evidenced in the empirical literature summarised here. Existing, longstanding concepts of ‘good’, ‘bad’ or ‘unpopular’ patients (e.g. Kelly & May, 1982; Stockwell, 1972) are relevant, but could be improved by using an interactionist conceptualisation of the nurse–patient relationship, where patients who confirm the role of the ‘caring’ nurse are labelled by staff as ‘good’ (Kelly & May, 1982). Whilst there has been a recent turn in nursing theory from positivism to constructionism and more recently symbolic interactionist conceptions (Evans, 2016) Goffman's work remains relatively underused, particularly as it relates to constraints on patient agency and involvement in nursing care.

Shattell (2004) makes a compelling case for the greater use of Goffman's theory of face work in research into nurse–patient interactions, which has particular use in exploring how the ‘good patient’ role might limit the negotiation of care in nursing staff‐patient interactions (e.g. Maben et al., 2012). More recently, Featherstone and Northcott (2020) and Bridges et al. (2020) referred to Goffman's work on the Total Institution in understanding the care of older people and people living with dementia when in hospital. The empirical literature reviewed above demonstrates how elements of the Total Institution, such as withholding of information and depersonalisation—where the patient is no longer acknowledged as a person (Goffman, 1961)—are likely to be helpful in conceptualising and understanding poor care. The tension between humane individualised standards and the ‘batch living’ of institutional efficiency described in the Total Institution can also help us explore the impact of structural constraints on staff (Goffman, 1961). In this conceptualisation, as in the literature outlined above, patients who make fewer requests are preferable (Goffman, 1961). This provides some conceptual support to the idea of how difficult patients are managed (e.g. Maben et al., 2012), where patients have a moral responsibility for their actions and staff use privileges and punishments to manage ‘good’ and ‘bad’ behaviour (Goffman, 1961). Patients aim to ‘stay out of trouble’, which ‘is likely to require persistent, conscious effort in terms of the line presented’ (Goffman, 1961: 43). A line is ‘a pattern of verbal and nonverbal acts by which he expresses his view of the situation’ (Goffman, 1967: 5), which ‘tends to be of a legitimate institutionalised kind’ of a limited array of options (Goffman, 1967: 7). When lines are internally consistent and believed by others, people maintain ‘face’: ‘an image of selfdelineated in terms of approved social attributes’ (Goffman, 1967: 5). The presentation of self (i.e. one's line) is observed by others to assess genuineness (Goffman, 1959). If there are discrepancies, it can be discredited, meaning people are ‘out of face’. When in ‘front stage’ areas, performers—including nursing staff performing to patients (Goffman, 1961)—are expected to maintain this performance, which can be dropped once ‘back stage’ with one's team (Goffman, 1959). People are expected to make efforts to save the face of others, particularly if those others have more power or prestige (Goffman, 1967)— like nursing staff (Goffman, 1961). This can include avoidance tactics on the part of the less powerful person, where this person might make ‘slight modifications of […] demands on […] others […] so that they will be able to define the situation as one in which their self‐respect is not threatened’ (p. 17)—which could include deciding not to flag missed care. If face is not saved, hostility might be encountered, as in the case of patients being seen as difficult by staff (Featherstone & Northcott, 2020; Maben et al., 2012). Taken together then, Goffman's work on the presentation of self, face work and the Total Institution could generate new insights into how patients navigate nurse–patient interactions.

The aim of this paper was to explore the patient role in nursing staff‐patient interactions relating to fundamental care omissions and whether face work, the presentation of self and the Total Institution might add to its conceptualisation.

## METHODS

These qualitative findings were taken from a wider study aimed at exploring the feasibility of increasing patient choice in fundamental care delivered in two hospitals in the south of England. This project was developed in response to a stakeholder consultation where communication, the provision of information and involvement of patients and their families/carers in care were identified as key elements involved in ‘good care’. The wider study was intended to measure the impact of a complex intervention to increase patient involvement in fundamental care decisions (see Bridges, Gould, et al., 2019) with an adapted version of the Tell Us card, a communication tool, which aimed to facilitate communication between nursing staff and patients and support patient participation in decisions (Jangland et al., 2012). The adapted version was agreed through a series of focus groups with staff and recent patients. Full details can be found here (https://doi.org/10.1186/ISRCTN38405571). Ethical approval was gained from the HRA (IRAS number 216598), with Research Ethics Committee approval received from London—Harrow Research Ethics Committee.

This paper is based on findings from three patient focus groups that ran in October and November 2017 and twenty interviews with patients from four inpatient medical and/or surgical wards in two general hospitals during February–April 2018. Interviews and focus groups were chosen to explore patients’ role in negotiating care, with a particular focus on the work patients undertake to negotiate the meeting of fundamental care needs with nursing staff (both nurses and healthcare assistants) in an appropriate way.

Six people were recruited to three focus groups through an email sent to all members of a local research group database of people interested in contributing to healthcare research. This followed unsuccessful attempts to recruit from information shared in discharge waiting rooms in the hospitals and posts on one hospital's Facebook page. Inclusion criteria were that people were required to have had at least one overnight hospital stay within the last 2 years. People were paid for their time and expenses for participating. The aims of the focus groups were to explore patients’ experiences of fundamental care in hospital and to co‐design a patient involvement card for use in the main intervention study. Focus groups were facilitated by JH and LS and used a semi‐structured topic guide. Each focus group was 1 h long. The first focus group involved discussing experiences of receiving fundamental care in hospital, experiences of poor and good care, involvement in decision‐making around fundamental care, responses to requests to have care delivered differently, staff reactions and how they felt involvement in care decisions could be improved. All data from this focus group was included in the analysis for this paper. Whilst demographics were not collected, participants included men and women of working age as well as retirees. Participants had been hospitalised for at least two nights, for different total numbers of visits and for different reasons within the last 2 years.

For inpatient interviews we aimed to build a realistic maximum variation sample (across age, gender and time spent in hospital) given that the hospital population differs from the general population and by clinical specialty. Five patients from each of four intervention wards in the wider study were recruited to interviews (*n* = 20). Researchers were provided with a full list of patients from the Research Nurse team on the day before their visit to a ward and aimed to recruit at least one and up to three participants per ward visit. On reaching the ward, the researchers asked staff which patients would be unable to communicate their choices about taking part in the interviews, who was unconscious or if there were clinical concerns that precluded recruitment. These patients were excluded from the patient list. Researchers then approached each non‐excluded patient to describe the study and share study information. If a patient was interested in and able to participate in the study that day, written consent would be taken, and interviews scheduled for the same day. Anonymised demographics were collected during interviews and saved on a locked, shared document. As interviews progressed, researchers would purposively sample patients to build a maximum variation sample as described above. As interviews took place in a private room, only patients in a single room or those able to walk to a private room close to or on the ward were able to participate. On one ward no private separate room was available, so all interviews took place in single rooms. Demographics of interviewees are shown in Table 1. There was good variation in terms of age, gender and length of stay on ward. Interviews explored experiences of fundamental care, personal meaning of ‘good care’, views on involvement and experiences of being involved (or not) in fundamental care choices. As ideas and concepts emerged during interviews and analysis, conducted iteratively, interviewers shared initial conceptual ideas, which were explored during subsequent interviews, and interviews were continued until data saturation was reached.

**TABLE 1:** Interviewee demographics

Demographics	*n* (=20)
*Gender*	
Female	12
Male	8
*Age*	
29 or under	2
30–39	3
40–49	4
50–59	3
60–69	4
70–79	3
80+	1
*Length of stay on ward*	
Overnight	1
2 days	1
3–4 days	6
5–7 days	5
Over 7 days	7

All interviews and focus groups were recorded and transcribed. To maximise recruitment and reduce patient burden on a busy ward where patients were anxious about leaving their bed, we agreed to keep interviews to a maximum of 30 min unless the interviewee felt able to offer more time. Excepting two shortened recordings due to recording errors, where total interview length was unknown, interviews lasted between 12 and 38 min, with the majority over 20 min long. Fifteen patients were interviewed by JH and five by LG.

All interview data, all data from the first focus group and relevant sections from the second and third focus groups were analysed together, although each manuscript clearly identified the source of the data. Findings were coded using the thematic analysis approach described by Lofland et al. (2006). This allows the combination of both bottom‐up coding and some top‐down organisation of coding to explore specific research questions. This involved a three‐level process, beginning with first order initial codes applied to all data, preserving participant wording. Then second order thematic codes were created to draw together initial codes (or promote them to second order codes) in an iterative process where disconfirmatory data were sought and codes were merged and separated to ensure the best fit between codes and data. Finally third order, theoretical coding was developed iteratively to bring thematic codes together, in this case to create an interaction typography by support need. One researcher (JH) carried out all the coding, with frequent discussions with all co‐authors to build consensus around thematic and theoretical coding. Finally, member checking was carried out by discussing impressions of staff and impact on patient experiences with a local stroke survivors’ group. Members recognised the different nursing impressions described here and the difficulty of asking for person‐centred fundamental care whilst in hospital.

## FINDINGS

Like patients in Total Institutions, interviewees described a preoccupation with ‘staying out of trouble’ (Goffman, 1961: 43) through ‘not wanting to make a nuisance’, ‘pester’ staff or ‘upset the applecart’, protecting the face of ‘good patient’ through the line taken with staff. Participants described their continual work to test the veracity of (or ‘discredit’) nursing staff's performances as ‘caring’. When nursing staff's lines were convincingly commensurate with ‘caring’, patients believed staff were invested in their wellbeing. When nursing staff appeared ‘available’, patients believed they were able to make time for personalised care and requests for care would not threaten staff face.

In protecting the faces of both patients and staff, interviewees described assessing nursing staff lines to judge whether their face as a ‘good patient’ was likely to be compromised by requesting care, when and how they would ask, and if care requests were likely to be honoured. Nursing staff seen as ‘engaged’ presented a convincing face of being both attentive and caring. Those who were perceived as ‘distracted’ were convincing as ‘caring’, but not as ‘attentive’. Finally, nursing staff whose face was not convincing to patients in terms of being either caring or available were seen as ‘dismissive’ of patient needs, and care was experienced as dehumanising, with patient requests routinely ignored.

The wards in the study had large ‘front stage’ areas (Goffman, 1959), with limited ‘back stage’ areas where nursing staff could suspend their performance (sluice rooms, medicine cupboards, small staff rooms). During researchers’ time on the wards, the nursing team were often ‘front stage’ even when not directly attending to patients, for example completing paperwork on desks within patient bays, or at the front desk. This meant it was challenging for nursing staff to consistently appear available in their performance to patients, especially when workload was high. A staff member's ability to present a face as both available and caring (‘engaged’) appeared to be compromised by task‐led nursing, high turnover of patients and whether patients were admitted with a pre‐existing long‐term condition. For instance, on one ward (A1) there were comparatively few members of staff described as ‘engaged’. This ward had a relatively high turnover of patients and number of beds and was undergoing a significant reorganisation during data collection. These factors were likely to decrease staff capacity in relation to workload, reducing their capacity to maintain lines consistent with a ‘caring’ and ‘available’ nurse and to provide fundamental care to an acceptable standard to all patients. The ward where interviewees most consistently described staff as ‘engaged’ was a transplant ward, where overall length of stay was longer, and patients with related chronic problems had existing relationships with many members of staff in the specialty area.

Perceived interactions from a single ward tended to weight more towards one category, or appear to overlap slightly, suggestive of a continuum of care with engaged at one end and dismissive at the other. Interviewees and focus group participants noted that on wards where not all staff were perceived as engaged, they would wait to make fundamental care requests from staff presenting as ‘engaged’, waiting until the next shift if necessary. However, some patients described feeling that all staff were ‘out of face’, meaning they were unwilling to risk their and the staff's face by asking for help and potentially risking hostility (following Goffman, 1967). In some cases patients felt so dehumanised that they stopped asking for care because they felt it would not be given.

### Engaged—‘Nothing is too much trouble’

Interviewees described feeling able to request care from ‘engaged’ staff whose performances were consistent with the caring and available nurse. The staff line described by patients was of nursing staff actively encouraging patients to make fundamental care requests (including using call bells) and made clear efforts to personalise patients’ fundamental care. Nursing staff would take time to chat or joke with patients and to find out something about them as a person. Interviewees described making close observations of nursing staff's performances as caring and available, picking up on subtle discrepancies in the line presented.

it sounds genuine whereas sometimes it's sort of like, 'Are you okay?' and they walk off before…yes. They don't seem to do that on B2. They actually seem to listen (Interview 17, Ward B2).

When staff were assessed to be genuinely engaged (maintaining face as both available and caring), patients could ask for care without this compromising their face as good patients.

it makes a difference. You don't then feel that you're a nuisance, or a bind, and you're not upsetting the applecart (Interview 11, Ward B1)

Patients perceived staff to be genuinely ‘engaged’ when they saw them making time to respond to requests, even when there were many patients to support.

I thought: You've got a really full bay here, yet nothing is too much trouble. (Interviewee 2, speaking about a non‐study ward)

Patients who were less able to carry out fundamental care were as reluctant as other patients to threaten their own face as a ‘good patient’ by using their call bell, even with encouragement from staff.

They're like, 'No, we want you to. That's our job. […] so I don't feel quite so bad now. (Interview 6, Ward A2)

‘Engaged’ nursing staff were observed to provide ‘inventive doctoring’ (Mol, 2008) as part of their line, including adapting standard pressure ulcer prevention techniques for a paraplegic patient.

one of the nurses has come up with a good solution now which is much better using something smaller, so we've compromised on that (Interviewee 7, Ward A2)

Staff perceived as engaged eschewed batch living approaches that prioritised routinised tasks.

I used to get a lot of pain *before* my drugs were due […] She'd go, 'Okay, I'll just go off and get the nurse.' (Interview 19, Ward B1).

### Distracted—‘they mean well but they are very busy’

Batch living approaches by some staff were evident to patients, who would not ask for help if they could see nursing staff were engaged in a routinised task and not presenting a line as ‘available’. This was the case even if the patient had restricted mobility or required support to accomplish fundamental care tasks. These members of staff were perceived as caring, but unavailable (‘distracted’).

I wouldn't say [it’s] easy [to talk to a nurse] because they're you know, they're in and out, aren't they? 'Just coming to do your blood pressure. Just coming to do that.' (Interview 16, Ward A2).

When patients perceived staff as too busy to be genuinely available, they tried to manage without support. This included patients with restricted mobility after a significant operation.

if I can get away with it I'll just lay here and do it all, and if I happen to see a nurse coming I just call her quickly (Interview 10, Ward A2).

Interviewees discussed this unwillingness to ask for care as a form of avoidance tactic so their ‘good patient’ face would not be threatened.

I'm not the type to keep pestering anybody. I'll put up with things for a long time before I need to call anybody. (Interview 10, Ward A2)

However, amongst patients who were able to mobilise alone, it could be unclear what they were ‘allowed’ to do for themselves without risking discrediting their own face, as with this patient who was explaining why she did not leave the ward to mobilise.

I don't know if it's confidence that I'd get lost, or whether I shouldn't be doing it, or if I'd get in trouble for doing it […] (Interview 11, Ward B1).

Personal care for patients requiring support could be missed, delayed or interrupted, but interviewees continued to present a line to maintain face as the ‘good,’ uncomplaining patient.

we did try and wash my hair […] two days ago I asked and the nurse that was going to do it ran out of time, which is ‐ it was *so* busy, and I understand that. They had lots of people messing and she was really apologetic (Interview 6, A2).

Patients requiring more support in care tasks reported sometimes feeling unable to ask for fundamental care that was missing from washing routines, although it was offered if asked for. There was a sense of needing to renegotiate care every day, and an unwillingness to do so, reflecting Maben et al.'s findings (2012).

the only thing I would say is teeth, cleaning teeth and some automatically when you're having your wash […] get the bowl and it's just part of the routine, but others, obviously I've got a tongue in my mouth, I can ask, you know, which I usually do say, 'Can I do my teeth' and it's not a problem. But I think there were a few days at the beginning when I didn't get them done and I didn't ask (Interview 7, Ward A2).

As part of their examination of staff performances, patients noticed when care for other patients was inadequate.

He don't eat chicken. […] They put a note up above his bed [saying he says ‘yes’ to everything] and it's like all agency people that's coming in, yes, and they're just going, 'Roast chicken', they're going, 'Ching' [mimes ticking box], like that and they're not looking at that (Interview 1, Ward A1).

### Dismissive—‘the nursing staff don't listen to you’

Staff who were ‘distracted’ were seen as caring though unavailable due to their workload, and patients would work to protect staff face. However, ‘dismissive’ staff were perceived as deliberately withholding their time and attention from patients.

They're busy, I know, but they could manage more. They yak, they talk too much, (Interview 12, Ward B1).

They just sort of come round and do things and then walk off; instead of sort of asking you what you'd like (Interview 3, Ward A1).

Here the relationship between staff and patients was entirely limited to the batch living tasks of a Total Institution. Tasks were carried out with the minimum of interaction and care was arranged around the needs of the institution (or staff) rather than patients. Choices were limited or absent, with patient requests denied without explanation. This resulted in depersonalisation: feeling dehumanised, angry or infantilised.

we had lights off the other night at quarter to nine, which is obviously a bit too early. […] It felt like being a child and having to go to bed at night. (Interview 3, Ward A1).

I got told to go and have a shower this morning ‐ that was quite funny […] She said to me, 'Go and have your shower now.' 'What?' I said […] 'No, I'll go later. Don't worry. I had one yesterday. I don't smell that bad.’ (Interview 2, Ward A1)

They've been taught to put the pillow across both feet, keep the heels off the [bed] […] I said, 'I don't want that.' Well, they said, 'Well, this is the way we do it, you know.' […] Well, when they go, I change it. I don't argue with them (Interview 8, Ward A1)

As a result, even patients able to self‐care described how they were discouraged from carrying out care for themselves.

it definitely does make you feel slightly like ooh yeah, like I'll just leave it ‐ which, actually, I did last night, because I wanted some more water and they told me to go and ask them rather than just go in the kitchen and get it ‐ and they were all talking […] so I just went back and waited until I saw someone walk past (Interview 2, Ward A1).

There was also evidence of the withholding of information characteristic of a Total Institution, even if the patient possessed medical knowledge, as in this example.

I […] just wanted to know what my saturations were like […] and she said, 'Oh I don't know. I've logged it now,' and she just walked off. […] (Interview 3, Ward A1).

For patients who were frail or confused, routinised care could be particularly poor and delivered in a way that compromised their dignity and expressed choices.

last night, I had some chocolate buttons […] and I asked [a frail, ‘muddled’ patient] if she wanted one [in the morning]. And she was like, 'Yes, yes.' So I said to the staff member this morning: 'Can you give one to her? She wants it.' She went over and she went, 'Oh, don't worry, she'll have forgotten that she asked you for one.' I was like, 'You're saying that in front of everyone; and even if she has forgotten, ask her if she wants one now.' She said, 'Oh, don't worry, dear, she forgets.' And then before her wash she said, 'I'm just shouting really loudly just to let everyone know I'm going to do the wash; because she's going to put a complaint in about us, otherwise, because we're not washing her.' (Interview 2, Ward A1)

Patients requiring support with mobilisation also received poorer care, with call bells left unanswered for a long time and fundamental care interrupted, leaving patients vulnerable.

she was left mid‐wash because another lady was being discharged and needed to be hoisted. But transport wasn't actually here yet, so that could have actually waited until the end of the wash ‐ which I actually think is quite rude: unless there's an emergency, you really shouldn't be leaving someone […] half naked on a bed to get chilly (Interview 2, Ward A1).

This reflected existing research that has documented how the treatment of more vulnerable patients—who require greater input and whose care is considered intrinsically less ‘rewarding’—can be perceived as difficult by nursing teams, resulting in substandard care (Featherstone & Northcott, 2020; Maben et al., 2012).

Interviewees described how patient suffering was ignored—another hallmark of dehumanisation.

she was really coughing, really hard, and coughing up quite a lot of stuff; and one of the staff just walked past her, looked at her […] Didn't say anything. […] clearly, when you've just sauntered in and sauntered out, you do have two minutes to sit there… (Interview 2, Ward A1).

the male nurse came up to me to take my blood pressure, I said, 'I'm on painkillers.' And he totally blanked me, totally. And of course, then he walked away without talking to me and I screamed at him, 'Help me,' you know. And then another nurse came along, and I said to her what he just done. She said, 'He's taking your blood pressure.' […] I was given it soon after that […] I made a racket first though. (Interview 8, A1).

Ultimately, as in this case described in a focus group, the sense of dehumanisation could be so strong that even patients able to mobilise and requiring further medical treatment felt they had no option but to leave the ward.

You sort of feel an expectation of contact and then it's gone ‐ and then that made me feel worthless. I described it to my surgeon as feeling like a piece of meat on the slab and there were occasions when that was reinforced on the ward because I ceased to be a person. I didn't have a name anymore; I was just a bed, I was a body in a bed that needed things doing to it. […] I ended up making that decision and not telling them that I had an infected wound. Nobody checked it and nobody asked so I didn't tell them because I desperately wanted to go home (‘Roger’, second focus group)

### Maintenance of face and support needs of patients

Important distinctions emerged between patients who had greater physical autonomy and were recognised as possessing mental capacity, who were able to protect their face more easily through carrying out their own fundamental care where possible. Where staff maintained face as both available to patients and caring (engaged), patients received care that was appropriate to their needs and preferences, regardless of level of physical dependence on nursing support (see Table 2). However, we did not have enough data to explore whether this level of personalised care was available to patients who had difficulty managing their own face due to communication issues, as it was based on what interviewees told us about the care of proximate patients.

**TABLE 2:** The impact of engaged, distracted and dismissive interactions on patient care by patient mobility and communication needs

Patient mobility and communication	Nursing staff maintain face as both available to patients and caring (Engaged)	Nursing staff maintain face as caring but lose face as available to patients (Distracted)	Total Institution—batch living, depersonalisation & withholding of information (Dismissive)
Patient unable to make a clear and direct request (communication difficulty/cognitive impairment) (This was through participants' reports of their observations of other patients' care).	*We did not have data giving examples of ‘engaged’ care being given to patients who were ‘confused’ or had evidence of a cognitive impairment*.	Care notes about communication, for example ‘this patient says yes to everything’ are not noticed by staff, and inappropriate care is given (e.g. food a patient does not eat).	Where an advocate (including other patients) makes a request, this may be dismissed by staff (‘'Oh, don't worry, she'll have forgotten’).
Patient requiring staff support for all or most care (unable to move from bedside), without cognitive impairment.	Patients reluctant to use buzzer to ask for help begin to do so when specifically encouraged by staff.	Basic care provided but risk of this being inappropriate, delayed or with significant elements missed. Patients may feel unable to mention that care has been missed, protecting both staff and patient face.	Patients may experience delays when using their buzzer or care may be interrupted.
Patient with restricted mobility (e.g. able to leave bedside but with limited ability to move around independently)	Staff will respond to requests where feasible or explain why not. Staff may offer additional choices patients not initially aware of or problem‐solve to find a new solution outside ‘standard care’.	Patient will attempt to do as much for herself as possible and wait for staff to appear available in order to preserve staff and patient face. Care may be interrupted and left incomplete.	Patients' requests may be ignored; patient may employ subtle resistance (e.g. removing pillow from under heels) when staff members leave. Patients may feel they have to shout or scream when staff leave the room without, for example, providing the pain‐relief they requested.
Mobile patient	Patients are regularly asked if they need anything, feel comfortable asking and know they will receive what they ask for, even when it is busy.	Patients are able to manage largely for themselves (e.g. shower). Patients may be uncertain what they are ‘allowed’ to do or unaware of full range of choices, so may limit some activities to maintain a ‘good’ patient face.	Patients will provide as much of their own care as they can and can reject attempts by staff to direct care tasks they can complete independently. However, where care is controlled by the nurse, requests may be ignored, or information withheld. Patients may seek early discharge.

Note

Red squares indicate missed fundamental care, amber those where care omissions are mitigated by the patient, green where personalised care is received.

When staff maintained face as caring, but not as available to patients (‘distracted’), nobody reported receiving care appropriate to their needs and preferences. Instead the ‘batch living’ needs of the ward and hospital predominated. Patients who had full or restricted mobility were able to protect their line through providing some of their own care or delaying it without experiencing fundamental care omissions. However, they could be uncertain about what they were ‘allowed’ to do, which led to them restricting their own self‐care activities, so they didn't ‘get into trouble’. Crucially, patients who required nursing support to carry out fundamental care were unlikely to threaten their face as a ‘good patient’ by asking for what they saw as non‐urgent support or complaining if care was delayed or interrupted. Patients who had difficulty communicating their needs verbally to staff were at risk of fundamental care omissions, such as failing to get enough nutrition because communication needs were not supported.

Patients interacting with ‘dismissive’ staff had a qualitatively different experience, which was mostly immune to patient attempts to mitigate missed care. Care had the depersonalisation, information withholding and batch living constraints characteristic of the Total Institution. Patients described timely pain medication and information about their condition being withheld and experienced depersonalisation. Patients with limited mobility were at high risk of receiving inadequate fundamental care from these staff. They experienced delays in responses to call bells, or had care interrupted, where they could be left in undignified, unsafe situations and have their choices dismissed.

## DISCUSSION AND CONCLUSIONS

This paper aimed to explore the patient role in nurse–patient interactions relating to fundamental care, and whether face work, the presentation of self and the Total Institution might add to the conceptualisation of patients’ work in managing missed care. Patients described working to protect the face of ‘distracted’ staff (unavailable, but still ‘caring’), which meant they might not flag care omissions and would undertake care themselves (if they could) to mitigate this loss of care from nursing staff. However, for people with higher support needs who could not mitigate their own care omissions this could lead to a lack of nutrition, poor oral hygiene and other issues that could have serious consequences over a hospital stay.

Dismissive care was qualitatively different from both ‘engaged’ and ‘distracted’ care in that even patients with greater mobility and no cognitive impairments had limited power to challenge poor care. Instead, dismissive care was most closely identifiable with aspects of the Total Institution and easier to recognise as clearly substandard care highlighted in enquiries into patient care failings (e.g. Francis, 2013). Interactions with dismissive nursing staff were dehumanising, with care requests routinely ignored, demonstrating the limitations of patient face work to negotiate care omissions. This included assumptions of a lack of capacity for patients with cognitive impairments to make choices or failing to acknowledge capacity and communication issues in decision‐making. This reflects existing findings that show the UK Mental Capacity Act can be poorly understood in terms of assessing patient capacity to make decisions or involving suitable advocates to make ‘best interests’ decisions in hospital care (Heslop et al., 2013; Michael, 2008; National Patient Safety Agency, 2004; Tuffrey‐Wijne et al., 2014).

However, despite the clear distinction between ‘distracted’ and ‘dismissive’ care, patients who were most in need of physical support and patients with cognitive impairments experienced serious omissions of care in both cases. Our analysis also helps explain how missed fundamental care and care inequalities can happen in settings where staff are viewed by patients as outwardly caring. This contributes to understanding how and why patients with complex needs and cognitive impairments are particularly disadvantaged in acute hospital settings, and why they experience higher levels of mortality with reduced staffing.

These research accounts can only partially represent the total experience of the wider patient population. People in our focus group were involved in patient advocacy work, sometimes after experiencing particularly poor care, but had also experienced and could describe engaged care interactions. Our interviews were relatively short but limiting overall interview time enabled us to recruit a diverse sample of patients. The overlap of interview and focus group findings and positive member checking suggest good transferability (Lincoln & Guba, 1985) to a wider range of acute settings.

Interview data can only ever be a situated, social account rather than a direct reflection of experience, particularly when discussing others’ behaviour. Our interviewees presented their lines as ‘good’ patients or patients willing to discredit their face in the interviews as much as in the accounts they gave of their care. However, understanding patients’ work to present themselves as ‘good patients’ (including within interviews)—and the limits of this work in the face of perceived dehumanising approaches by ‘dismissive’ staff—has provided some useful insights into why person‐centred fundamental care is so difficult to establish in the acute hospital setting. Future work would do well to combine observational data with interviews to explore this topic further.

Exhortations to improve fundamental care and actively involve patients should reflect the complex interactions that can discourage patients from expressing care needs, even when staff appear caring, but are ‘distracted’. Performance measurement focussing on fundamental care, advocated by some (Feo & Kitson, 2016) could lead to an increased focus on task driven care in which nursing staff are distracted by the requirements of batch living, with standardised routines adopted by staff to efficiently manage demand in the face of staff shortages. Our findings indicate that such reinforcement of the Total Institution in acute health care may undermine the role of patients as active participants and still leave the most vulnerable patients at high risk of care inequalities and missed fundamental care.

To date policy and nursing literature exploring nurse–patient interactions has rarely engaged with sociological work. This means little consideration has been given to how structural constraints, health inequalities and power dynamics impact on patient agency. This paper demonstrated how a more coherent use of Goffman's work on the presentation of self, face work and Total Institution generates new insights into how patients navigate nursing staff‐patient interactions. Where staff could not maintain face as ‘engaged’, patient work to protect both staff and ‘good’ patient face restricted their ability to request personalised or missed care. Our work also adds to the sociological conceptualisation of care inequalities by highlighting how patients with complex needs are less able to mitigate care omissions by carrying out their own care discreetly as a way of preserving staff face. This shows how the unequal power dynamic between staff and patients is likely to have the greatest adverse effect on the health outcomes of the most structurally disadvantaged patients. Our findings are evidence that far from being a relic of the past, many elements of the Total Institution survive in modern healthcare settings despite policy drivers and staff attempts to support more individualised approaches to care. Unless nursing staff can maintain face as ‘engaged’ (despite organisational constraints that can reduce their capacity to do so) patient involvement in hospital care decisions will remain at the level of rhetoric.

## AUTHOR CONTRIBUTIONS

**Joanna Hope:** Conceptualization (supporting); data curation (lead); formal analysis (lead); funding acquisition (supporting); investigation (equal); methodology (equal); project administration (equal); validation (equal); visualization (lead); writing – original draft (lead); writing – review and editing (lead). **Lisette Schoonhoven:** Conceptualization (lead); funding acquisition (lead); methodology (lead); project administration (lead); supervision (lead); validation (supporting); visualization (supporting); writing – original draft (supporting); writing – review and editing (supporting). **Peter Griffiths:** Validation (equal); visualization (equal); writing – original draft (supporting); writing – review and editing (equal). **Lisa Gould:** Investigation (equal); project administration (equal); validation (equal); visualization (supporting); writing – original draft (supporting); writing – review and editing (supporting). **Jackie Bridges:** Conceptualization (equal); methodology (supporting); project administration (equal); supervision (equal); validation (equal); writing – original draft (supporting); writing – review and editing (equal).

## DATA AVAILABILITY STATEMENT

Data from this study is not available as participants did not give consent for data to be shared outside the research team.
